# Submicroscopic and Asymptomatic Congenital Infection by* Plasmodium vivax* or* P. falciparum* in Colombia: 37 Cases with Placental Histopathology and Cytokine Profile in Maternal and Placental Blood

**DOI:** 10.1155/2017/3680758

**Published:** 2017-03-28

**Authors:** Olga María Agudelo-García, Eliana María Arango-Flórez, Jaime Carmona-Fonseca

**Affiliations:** Grupo Salud y Comunidad-César Uribe Piedrahíta, Universidad de Antioquia, Carrera 51 D No. 62-5, Piso 3, Oficina 336, Medellín, Colombia

## Abstract

*Problem*. Congenital plasmodial infection (CPI) is a rare event, which has been little studied in Colombia.* Objective*. To measure the frequency of CPI and to describe the immune and histological characteristics in maternal blood and placentas when CPI occurs.* Methodology*. A cross-sectional study was carried out in northwest Colombia. A sample size of 39 unit analysis (a unit of analysis corresponds to the cord, placenta, and peripheral blood of a pregnant woman) was calculated using epidemiological and statistical parameters. Thick blood smear (TBS) and quantitative real-time polymerase chain reaction (qPCR) were used as diagnostic tests.* Results and Conclusions*. A total of 137 parturient women were studied. All cases of CPI were submicroscopic (TBS negative and qPCR positive) and asymptomatic infections. If the definition of CPI considers only detection of parasites in umbilical cord blood, regardless of what was found in peripheral or placental blood, the frequency of CPI was 27%. However, if that definition is stricter and includes simultaneous detection of parasites in maternal or placental blood with the same species, the frequency of CPI in this study was 13%.

## 1. Introduction

Plasmodial infection during pregnancy (gestational plasmodial infection) may or may not be accompanied by clinical signs and symptoms. Gestational infection is usually associated with placental plasmodial infection (parasites detected in placental blood or in placental tissue) and much less frequently associated with congenital infection [1]. Congenital plasmodial infection (CPI) can be detected in umbilical cord blood at delivery or in peripheral blood of the newborn until 30 days after birth; this limit of 30 days is arbitrary but frequently used [2].* Plasmodium vivax *and* P. falciparum *are the species most commonly associated with CPI. When the presence of parasites in blood (umbilical cord blood or neonate peripheral blood) is associated with clinical signs or symptoms, it is known as congenital malaria. This congenital infection is referred to as an infection acquired in utero and it must be differentiated from infection acquired after birth by a mosquito bite.

In general, there is no consensus on the definition of CPI or congenital malaria; some authors defined CPI when asexual parasites are detected in peripheral blood of the neonate within a week after birth [3]; others indicated CPI if* Plasmodium *spp. are detected in umbilical cord blood or in peripheral blood of the neonate within 21 days of birth, with or without clinical symptoms [3]; but other authors suggested that detection of plasmodial parasites in umbilical cord blood or neonate peripheral blood must be accompanied by detection of the same plasmodial species in maternal or placental blood to define a case of congenital malaria [4, 5]. In addition, a summary of criteria to confirm the intrauterine transmission of parasites in a neonate with umbilical cord blood or peripheral blood positive for* Plasmodium* spp are as follows: (a) no possibility of a mechanism of transmission different from the transplacentary route (e.g., vectorial transmission); (b) a history of gestational malaria caused by the same species detected in the neonate; (c) in cases where the mother lives in a non-malaria-endemic area, with travel history to a malaria-endemic area during pregnancy or transfusion during pregnancy or childbirth [4].

Clinical manifestations of CPI depend on the level of malarial immunity of the mother and the transplacental passage of antibodies [6, 7]. Although plasmodial parasitemia is more frequent in primigravid than in multigravid women, the converse is true in their offspring, especially in those with placental infection; newborns of primigravid women with placental infection had a lower risk of parasitemia, while placental infection in multigravida women was a strong risk factor for parasitemia during infancy [8].

In general, CPI is considered a rare event, but the frequency is variable in different regions; in sub-Saharan Africa, for example, the reported prevalence of CPI varies from 0 to 46% [9]. A compilation of published studies on the frequency of CPI in different African countries and in Colombia is shown in Table 1. A general conclusion is that CPI varies widely among countries and within the same country. Specifically in Colombia, the problem of CPI has been very little studied and the few studies found are restricted to the same region, the departments of Antioquia and Córdoba [10–13] (Table 1). Those studies indicated a prevalence of congenital infection in Colombia that varies from 0 to 2.7%, for a weighted average of 1.8%. Other authors have reported specific clinical cases of congenital malaria in Colombia [14, 15].

As part of a larger clinical study on pregnancy-associated malaria which has been developed in two municipalities in Antioquia and Córdoba, the present study aimed to measure the frequency of CPI and to describe the immunological profile in maternal blood and histological characteristics of the placentas of those newborns with CPI.

## 2. Methods

### 2.1. Study Site and Population

This study was carried out in northwest Colombia in the municipalities of Tierralta and Puerto Libertador (Córdoba). The malaria-endemic region is known as Urabá-Altos Sinú/San Jorge-Bajo Cauca, the departments of Antioquia and Córdoba, which has an estimated area of 43,506 km^2^ and a malaria at-risk population of 2.5 million distributed in 35 municipalities. The department of Antioquia reported 20,511 malaria cases in 2008, 32,029 in 2009, and 45,618 in 2010;* P. vivax* was reported in 65–70% of all cases. Meanwhile, in Córdoba, during 2001–2009, in 1,433,461 inhabitants, a mean of 37,616 cases/year was reported: 69% caused by* P. vivax*, 23% by* P. falciparum*, and 1% from mixed infections [16]. The municipalities of Valencia and Tierralta (southwest Córdoba, on the border with Urabá) and Puerto Libertador (southeast Córdoba) reported 90% of malaria cases in Córdoba [16]. Subjects for the current study were recruited at the local hospitals in Tierralta and Puerto Libertador [16].

### 2.2. Study and Sample Design

This study was part of another larger project to explore the epidemiology, clinical characteristics, and immunopathology of pregnancy-associated malaria in the Urabá-Altos Sinú/San Jorge-Bajo Cauca region in Colombia, during 2009–2014.

In order to measure the frequency of CPI, a cross-sectional study was designed. A sample size of 39 unit analysis (a unit of analysis corresponds to the cord, placenta, and peripheral blood of a pregnant woman) was calculated using the formula *n* = *NZ*^2^*p*(1–*p*)/[(*Ne*^2^)+(*Z*^2^*p*(1 − *p*))] [17], based on the following epidemiological parameters:

- *N*: reference population. A total of 1,352 live births were recorded during 2009 in Tierralta and Puerto Libertador, Córdoba, according to the National Department of Statistics of Colombia (DANE), as an approximation of the reference population, *N* = 1,352.
- *p*: probability of the event of congenital malaria reported previously by Carmona-Fonseca and Maestre-B in the same region, 2.7%, *p* = 0.27 [11].
- *Z*: confidence interval of 95%, corresponding to *Z* = 1.96.
- *e*: sampling error estimated in 5%, *e* = 0.05.

Pregnant women were recruited during labor at the obstetric facility of the official hospitals of Tierralta and Puerto Libertador, Córdoba. The plasmodial infection in maternal, placental, and umbilical cord blood was diagnosed using independent tests: microscopy (thick blood smear, TBS) and quantitative real-time PCR (qPCR). Microscopy was performed at the local hospital at time of childbirth. qPCR was carried out weeks later in Medellín.

Prenatal care for women, therapeutic decisions based on a particular situation of each woman, care delivery, and monitoring of the neonate were provided exclusively by the staff of the health institution in each locality. Researchers collected the biological samples from the mother, placenta, and newborn, performed the appropriate laboratory tests, and obtained the relevant data from hospital records.

### 2.3. Inclusion and Exclusion Criteria

The general inclusion criteria for the study population were as follows: >1 year of residency in the study region (Urabá-Altos Sinú/San Jorge-Bajo Cauca region); absence of serious general disease, complicated pregnancy, or complicated malaria; and informed consent. The only exclusion criterion was consent withdrawal.

### 2.4. Data and Specimen Collection and Preparation

Pregnant women who delivered at the local hospital were enrolled if their current residence was in a malaria-endemic community. Researchers obtained informed consent and completed a questionnaire with data on age, place of residence, number of pregnancies, and number of malaria episodes during the pregnancy reported by the mother and based on her health card that documents the diagnosis (by TBS) and treatment. Antenatal, labor/delivery, and infant outcomes data as well as maternal hemoglobin level at delivery were collected from the hospital chart.

Whole blood samples from the mother (peripheral blood), placenta, and umbilical cord were obtained in delivery rooms in the local hospitals. Maternal peripheral blood was collected by venipuncture. Cord blood was collected by sectioning a fragment to expose a fresh segment and then draining the blood into the tube, and fetal blood was collected without contamination from other maternal or fetal tissues including placenta and amniotic and allantoic fluids. After cleaning with saline, small (~1 cm^3^) sections of the placenta were removed from the maternal side, and the pooled blood was collected by pipette aspiration. Blood samples were collected in EDTA tubes (BD Vacutainer®, USA) and used to prepare thick blood smears and dried blood spots on filter paper (Whatman® Grade 3). Blood spots were prepared with approximately 100 *μ*L blood (two drops). After drying at room temperature, spots were sealed in plastic bags (one bag per sample per woman) and stored at 4°C. Total maternal blood was stored in TRIzol® for subsequent RNA isolation and analysis. For histological and apoptosis analysis, two pieces of placental tissue were obtained; one fragment corresponded to the area immediately next to the insertion cord (central fragment) and the other fragment corresponded to an area between the point of insertion of the cord and placental edge (middle fragment). Each fragment had a surface area of 3 × 3 × 3 cm through the entire thickness. Fragments were fixed in 10% neutral buffered formalin at room temperature and processed and paraffin-embedded within 48 hours at the histopathology laboratory in Medellín. Sections of 5 *μ*m thickness for each fragment were stained with hematoxylin and eosin and read under 100x and 400x magnifications according to standard procedures. Additionally, a tissue fragment was taken and stored in RNAlater® for subsequent RNA isolation and analysis.

### 2.5. Malaria Diagnosis

Field-stained thick films (TBS) were read by an experienced microscopist in the local research laboratory. Thick smears were defined as negative if 200 fields (100x magnification) were free of parasites. Parasite density was measured by counting the number of parasites per 200 leukocytes, based on a mean count of 8,000 leukocytes/*μ*L of blood.

For diagnosis by qPCR, an alcohol-sterilized hole punch was used to cut a circle (approximately 6 mm) from each filter paper, and DNA was extracted using the Saponin-Chelex method described by Plowe et al. (1995) [18]. qPCR was performed as described by Shokoples et al. (2009) [19]. Samples were first tested for* Plasmodium *using a genus-specific set of primers and hydrolysis probe (Plasprobe). Real-time PCR was performed on the ABI 7500 FAST platform, under universal cycling conditions. Samples with a Cycle Threshold (CT) value under 45 were tested in a duplex species-specific real-time PCR reaction for* P. falciparum* and* P. vivax* [19]. Parasite DNA concentration was quantified in the genus-specific screening reaction against a plasmid standard curve of known copy number included in each run. Concentrations are reported as the number of copies of the 18S rRNA gene per microliter of purified DNA. Most PCR assays target DNA of the* Plasmodium* multicopy 18S rRNA genes, which, due to their mosaic of conserved and variable regions and high copy numbers, provide a perfect molecular target for quantification and species identification. In our tests, we used amplification of the 18S rRNA genes from total DNA for quantification.

The most prominent molecular marker is the 18S rRNA gene, present at 5–8 copies per genome, depending on the parasite strain; this increases the sensitivity of qPCR using this gene [20]. Instead, it contains several single 18S-5.8S-28S rRNA units distributed on different chromosomes, with the sequence encoded by the rRNA gene in one unit differing from the sequences of the corresponding rRNAs in the other units [21]. Additionally, the expression of each rRNA unit is developmentally regulated, with different sets of rRNAs being expressed at different stages of the parasite life cycle [22]. Therefore, a copy of the 18S rRNA gene does not represent a single parasite; that is to say, a copy of the gene does not correspond to a parasite; a parasite may present one or more copies of the gene; therefore, they are not equivalent units.

### 2.6. Expression Analysis of Cytokines and Markers of Tissue Damage and Inflammation

Cytokines were measured in mRNA isolated from both maternal whole blood (in TRIzol) and placental tissue (in RNAlater), while markers of tissue damage, hypoxia, and inflammation were measured in mRNA isolated from placental tissue.

Relative quantitation for expression analysis was performed using a reverse-transcription real-time PCR assay (RT-PCR). Total RNA was extracted using QIAamp RNA Blood Mini® (QIAGEN) for the tissue and TRIzol reagent for the maternal whole blood. cDNA was synthesized using First Strand cDNA Synthesis® (Fermentas). The reaction was set up in a Roche LightCycler® or ABI 7500. The efficiency of the PCR reactions was determined based on mRNA extracted from a stimulated BeWo cell culture or peripheral mononuclear cells from a donor. Complementary DNA was serially diluted and expression of *β*-actin was used to normalize the assays using the delta delta Ct method (ΔΔCt) as described previously [23].

### 2.7. Quantification of Apoptosis

Apoptosis in placentas was assessed by a TUNEL assay as described previously [24]. Placental sections were deparaffinized and placed on Fisherbrand Superfrost Plus® slides. Detection of cells undergoing apoptosis was performed using the kit DeadEnd Colorimetric System® Promega TUNEL, according to the manufacturer's protocol. The apoptotic index was calculated based on the proportion of TUNEL stained cells observed in 10 fields (40x).

### 2.8. Histology

Histological analysis of decidua, intervillous space, and villi was based on specific variables established by the researchers. Two slides were prepared for each placenta, and each slide corresponds to each fragment (central and middle fragment). We read 20 fields per fragment; therefore, a total of 40 fields per placenta were analyzed. Data are presented as the mean value of each variable read in the 40 fields. Variables were identified as absent or present in each field (qualitative analysis) and according to their quantity in each field. For each variable, the absence of the event (value 0) was assigned when it was not found in any of the 40 fields. The presence of the event was measured by adding the number of events in each field and dividing the sum by 40 (mean value).

Variables evaluated in decidua were number of immune cells and number of fields with atherosis, necrosis, and abruptio. In villi, the variables were number of immune system cells, fibrin deposits, syncytial nodes, and number of fields with edema and infarction. In intervillous space, the variables were number of immune cells, number of fields with hemorrhage, thrombus, and calcifications. Other variables evaluated were number of villi per field, number of capillaries per villus, and presence, quantity, and location of infected red blood cells and hemozoin deposits. The definition of each histological variable evaluated is as follows [25]:

- Abruption: heavy hemorrhage observed in decidua.
- Atherosis: change in arteries of decidua, specifically the thickening of the arterial endothelium.
- Calcifications (placental calcification): calcium salt deposits in tissue, causing tissue degeneration.
- Capillaries/villus: number of capillaries in each villus.
- Decidual necrosis: ischemic area with degenerative lesions in decidua.
- Edema: fluid accumulation in stroma of the chorionic villus, characterized by expansion or swelling of the villus and presence of voids in the stroma.
- Fibrinoid or fibrinous deposit or deposition of fibrin: accumulation of fibrin in the villous stroma or around the villi.
- Hemorrhage: increase in red blood cells in the intervillous space.
- Immune cell: cells in decidua, villous, and intervillous space. The immune cells have nuclei that are deeply or densely stained (the chromatin is coarse and bulky) and almost fill the cells, with only a slight rim of cytoplasm around the nuclei. This cell may have a nucleus divided into two to five rounded or ovoid lobes that are connected with thin strands or small bands of chromatin. Syncytial nodes or knots: presence of small areas of terminal villi with thinning of the syncytiotrophoblast that covers them and thickening of the basement membrane of the trophoblast.
- Thrombus: blood clot as a result of bleeding.
- Villi/field: number of villi per field.

### 2.9. Other Definitions

Other definitions include the following:

- Malaria infection: a positive diagnosis by TBS and/or qPCR for* P. falciparum* and/or* P. vivax*.
- Congenital plasmodial infection (CPI): presence of* Plasmodium* in the cord blood not associated with clinical symptoms or signs.
- Congenital malaria (CM): presence of* Plasmodium* in the cord blood associated with clinical signs or symptoms. In infants in this study, monitoring was restricted to the period of hospitalization after birth, which is generally about 48 hours. Parents were asked to bring the child for medical consultation if symptoms appeared after leaving the hospital.
- Submicroscopic infection: a plasmodial infection positive by qPCR but negative by TBS.
- Anemia: hemoglobin <11 g/dL.
- Low birth weight (LBW): birth weight <2,500 g.
- Preterm delivery: birth before 37 weeks of gestation.

### 2.10. Statistical Analysis

Data were analyzed using EpiInfo® 6.0 and SPSS® 10.0. Kruskal-Wallis (K-W) and chi-squared (*χ*^2^) tests were used for comparison of continuous and categorical variables, respectively. Significance was set at *p* < 0.05. The following terms were used: mean, arithmetic mean; *N*, number of units; SD, standard deviation; SE, standard error of the mean; 95% CI, confidence interval of 95% of the mean; Min, minimum value; Max, maximum value; Lower L, lower limit of 95% CI; Upper L, upper limit of 95% CI.

### 2.11. Ethical Aspects

The study protocol was reviewed and approved by the (a) Ethics Committee of the Sede de Investigacion Universitaria (SIU), Universidad de Antioquia (Medellín, Colombia) (act number 07-32-126: project code 111540820495, contract: 238–2007, Colciencias), and the (b) Ethics Committee of the Instituto de Investigaciones Médicas, Universidad de Antioquia (act number 12: project code 111549326134, contract 611–2009, Colciencias, and project code 8764–2530, Regionalización, Universidad de Antioquia). Each participant gave full informed consent according to the Helsinki convention and the Colombian regulations for this type of research. Each subject voluntarily agreed to participate in the study.

## 3. Results

### 3.1. Demographic and Clinical Characteristics

A total of 141 parturient women were enrolled in this study, but 137 had complete measures in maternal peripheral blood, placenta, and umbilical cord blood.  Table 2 summarizes the demographic and clinical characteristics of women and neonates; there was no statistical difference in demographic and clinical variables in mothers or neonates, based on the presence or absence of plasmodial parasites in umbilical cord blood (congenital infection). In general, women were very young (23 ± 6 years old). The average parity was 2 ± 2; 26–37% were primiparous, 18–29% were secundiparous, and 55% were multiparous. Most women (82.5%) had a spontaneous delivery and 17.5% had a cesarean section. There was one stillbirth.

### 3.2. Frequency of Plasmodial Infection in Each Compartment

Based on microscopy, the frequency of plasmodial infection in maternal, placental, and umbilical cord blood was 8% (11/137), 3% (4/137), and 0%, respectively. The mean number of parasites/*μ*L was 2,787 ± 5,410 in maternal blood (range: 40 to 18,060 parasites/*μ*L of blood) and 381 ± 489 in placental blood (range: 40 to 944 parasites/*μ*L of blood).

Based on qPCR, the frequency of plasmodial infection in maternal, placental, and umbilical cord blood was 39% (54/137), 51% (70/137), and 27% (37/137), respectively. The mean number of DNA copies/*μ*L of reaction was 4,708 ± 18,453 in maternal blood, 1,250 ± 7,341 in placental blood, and ±22.0 ± 117.0 in umbilical cord blood; the maximum number of DNA copies/*μ*L in the cord was 713.

There were 37 neonates with a positive qPCR diagnosis in cord blood. None of these babies presented with clinical signs or symptoms during the first 24–48 hours while they remained in the local hospital. Moreover, in the clinical chart, there was not any other laboratory test for those neonates. In addition to being asymptomatic, all cases of plasmodial parasites in umbilical cord were submicroscopic since they were negative by thick blood smear.

The species distribution among the 37 positive results in cord blood was 70%* P. falciparum*, 37%* P. vivax*, and 3% mixed infection (positive for both species). Among the 70 positive results in placenta, the species distribution was 52%* P. falciparum*, 37%* P. vivax*, and 11% mixed infection; and among the 54 positive results in maternal peripheral blood, the species distribution was 48%* P. falciparum*, 50%* P. vivax*, and 2% mixed infection. In summary,* P. falciparum* was detected in 70% of all infections, but* P. vivax* was the predominant species in peripheral blood (50%), and mixed infections were very common in placenta (11%). Therefore, in cord, the* P. falciparum* :* P. vivax* : mixed infection ratio was 26 : 10 : 1, while in placenta it was 4 : 3 : 1 and in peripheral blood it was 26 : 27 : 1.

Highly significant associations (*p* < 0.001) were found in qPCR results per pairs of compartments (i.e., cord-placenta, cord-mother, and placenta-mother) (Table 3(a)):

- Cord-placenta: 45% (62/137) of subjects were negative in both compartments, 19% (26/137) had the same species in both compartments (69% (18/26)* P. falciparum*, 27% (7/26)* P. vivax*, and 4% (1/26) mixed), and 36% (49/137) had discordant results.
- Cord-mother: 50% (68/137) of subjects were negative in both compartments, 15% (21/137) had the same species in both compartments (67% (14/21)* P. falciparum*, 28% (6/21)* P. vivax*, and 5% (1/21) mixed), and 35% (48/137) had discordant results.
- Placenta-mother: 40% (55/137) of subjects were negative in both compartments, 27% (37/137) had the same species in both compartments (59% (22/37)* P. falciparum*, 38% (14/37)* P. vivax*, and 3% (1/37) mixed), and 33% (45/137) had discordant results.

Of the 37 positive cases in cord blood, 18 (48%) had the same plasmodial species in maternal and placental blood simultaneously (Table 3(b)) (#1, #3, and #7): 35% (13/37) with* P. falciparum*, 11% (4/37) with* P. vivax*, and 3% (1/37) with mixed infection). Moreover, 2 cases (2/37, 5%) had simultaneous infection in the three compartments (cord-maternal-placental) but the species were discordant; however, those discordant cases had mixed infection in placenta while in cord or maternal blood only one species was detected (#9 and #10). Four cases had discordant species among the compartments (#11, #12, and #13); three were positive in cord but negative in placental and maternal blood and a case was positive for* P. falciparum* in cord, positive for* P. vivax*, and negative in maternal blood. In summary,* P. falciparum *was detected in 70% (26/37) of cord blood positive samples; 70% (26/37) of cord blood positive samples had simultaneous and concordant species in placental or 57% (21/37) in maternal blood. Most of the discordant species across the compartments were due to mixed infection in the placenta, while a single species was detected in maternal or cord blood.

### 3.3. Frequency of CPI

**Figure 1:**
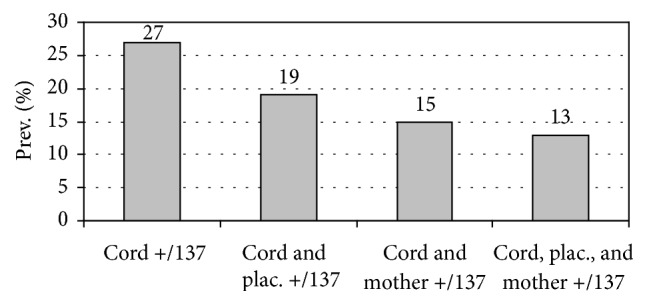
Prevalence of plasmodial infection in 137 cord-placenta-mother triplets, according to qPCR.

If the definition of CPI considers only detection of parasites in umbilical cord blood, regardless of what was found in peripheral or placental blood, the frequency of CPI in this study was 27% (37/137). But if that definition is stricter and includes detection of the same species of parasites in maternal, placental, and umbilical cord blood, the frequency of CPI of this study was 13% (18/137) (Figure 1).

### 3.4. Placental Histological Findings

A comparison of the placental histological findings in decidua, villi, and intervillous space, according to the presence of parasites in cord blood by qPCR, is presented in Table 4. Part (a) compares the frequency of each histological finding (Table 4(a)), and part (b) compares the quantitative measure for each variable (Table 4(b)). The frequency showed no statistical difference according to the presence of CPI in any of the sectors evaluated (decidua, villi, and intervillous space) except for edema in villi. In the quantitative evaluation, the only significant difference was a greater number of immune cells in decidua and villi when congenital infection was present.

The placental histological findings in the 18 newborns who were positive for the same plasmodial species in the three compartments (mother, placenta, and cord) were similar to the total group of 37 neonates with plasmodial infection in the umbilical cord.

### 3.5. Expression of Apoptosis, Inflammation, and Angiogenic Markers and Cytokines

The frequency of cells that underwent apoptosis was significantly higher in the presence of congenital infection (Table 5). In addition, the expression of FAS, COX1, COX2, HIF, VEGF, TNF, and TGF-b was significantly higher when congenital infection was present. All these variables were similar in the 18 newborns who were positive for the same plasmodial species in the three compartments (mother, placenta, and cord) and the total group of 37 neonates with plasmodial infection in the umbilical cord.

Six cytokines (immunomodulatory, proinflammatory, and anti-inflammatory) were evaluated in maternal blood and five had a significantly higher level of expression when plasmodial infection was detected in the cord. Only IL-4 had a similar expression between negative and positive congenial infections (Table 6). These findings were similar in the 18 newborns who were positive for the same plasmodial species in the three compartments (mother, placenta, and cord) and the total group of 37 neonates with plasmodial infection in the umbilical cord.

### 3.6. CPI by* P. vivax* versus* P. falciparum*

Although there were few cases of CPI per species, 10 for* P. vivax* and 26 for* P. falciparum*, in this section, we compared all the variables studied per species, as an initial approach to the problem. In summary, we only found a difference in expression levels of IFN-g and TNF in placenta: IFN-g was higher with* P. falciparum* (*p* = 0.001) and TNF was higher with* P. vivax* (*p* = 0.005). Comparison of the other variables (atherosis, necrosis, abruptio, edema, hemorrhage, thrombus, calcifications, fibrinoid deposits, syncytial nodes, villi/field, capillaries/villus, number of hemozoins per field, immune cells in decidua, cells in apoptosis, and expression of FAS, FASL, COX-1, HIF, VEGF, and cytokines IL-2, IL-4, IL-10, IFN-g, TNF, and TGF-b in placental blood and IL-2, IL-10, and TGF-b in maternal blood) was not statistically different according to the species of infection.

## 4. Discussion

The young age of women evaluated in this study (23 ± 6 years old) and relatively high parity for that age (26–37% were primiparous, 18–29% were secundiparous, and 55% were multiparous) agree with the previous findings in Colombia in the same region [11–13] and other continents [26–30]. However, it is important to note that in a study in three regions of Nigeria the average maternal age was 29.0 ± 5.1 and parity was 1.96 ± 1.76 [3]. These data indicate that women studied here had a higher gestational precocity. In addition, the average age of mothers of this study was considerably lower than the age found by Falade et al. in four cities and states of Africa: 23 versus 29 years [3].

The low frequency of stillbirth of this study (one stillborn in 137 newborns) is also consistent with previous reports based on more than 200 births in malaria-endemic municipalities of Northwestern Colombia [11, 13]. Apparently, plasmodial infection did not cause premature birth, as the pregnancy ended around week 37-38, on average. As usual, in Colombian pregnant women in malaria-endemic areas, a high percentage of mothers had hemoglobin levels lower than 12 g/dL [11, 31–33], and this is consistent with reports from other countries [3, 26–28, 30]. It is important to note that infants with CPI had less than 107 grams of body weight, compared with those without CPI. This has been a repeated finding in Colombian studies. Although the frequency of low birth weight (newborns weighing less than 2,500 grams) is very low, when plasmodial infection is present, the birth weight is lower, compared with those newborns without plasmodial infection [11, 32, 34].

The level of infection in cord blood was very low in this study; microscopy always was negative, and, by qPCR, the mean number of DNA copies was only 22 ± 117 copies/*μ*L. Also, Falade et al. found a low level of parasitemia in neonates, based on microscopy (48 asexual forms/*μ*L, range: 8–200/*μ*L) [3]. Maternal and placental parasitemia were the most important risk factors for patent neonatal parasitemia (*p* < 0.0001) according to Falade et al. [3], and similar findings were observed in our study. Age, parity, gestational age, positive history of malaria in pregnancy, and birth weight were not associated with CPI. However, of 54 women in labor with peripheral blood positive for* Plasmodium*, 39% (21/54) had newborns with a positive diagnosis in cord blood (CPI), while only 19% (16/83) of women negative for peripheral parasites had neonates with CPI. In addition, of 70 placentas positive for* Plasmodium*, 46% (32/70) were from newborns with CPI, while only 7% (5/67) of placentas positive belonged to neonates with CPI.

These support the importance of antenatal consultation for women residing in malaria-endemic regions including at least a thick blood smear test at each antenatal visit, regardless of presence of fever or malaria symptoms. Moreover, there should be consideration in Colombia on the use of intermittent preventive treatment for pregnant women (IPT-g), which has demonstrated beneficial effects and almost no harm in pregnant women in sub-Saharan Africa [35].

All newborns with CPI detected in this study had asymptomatic and submicroscopic infections, which is consistent with the low level of DNA copies. These two characteristics (asymptomatic and submicroscopic) are observed frequently in plasmodial infections detected during pregnancy in this region of Colombia (Urabá-Altos Sinú/San Jorge-Bajo Cauca, Northwestern Colombia), regardless of the compartment (peripheral maternal blood [gestational malaria], placenta [placental malaria], and cord blood [congenital malaria]) [13, 32, 33]. However, it is important to remark that some studies have shown the pathogenic role of those plasmodial submicroscopic infections during pregnancy [36].

The frequency of plasmodial infection in maternal, placental, and cord blood detected by thick blood smear was 8%, 3%, and 0%, respectively, which is similar to previous reports in the same region [11–13, 31]. But using qPCR, the frequency of plasmodial infection increased significantly, to 39% in maternal blood and 51% in placental blood, which is also consistent with previous reports [11–13, 31]. The frequency of congenital infection was zero by microscopy but ranged from 13% to 27% by qPCR depending on the diagnostic criteria applied: (a) considering only detection of parasites in umbilical cord blood, it was 27%; (b) considering detection of parasites in both placental and cord blood, it was 19%; (c) considering detection of parasites in both maternal and cord blood, it was 15%; (d) considering detection of the same species of parasites in maternal, placental, and cord blood, it was 13%. We think that, in this study, the finding of* Plasmodium* in cord blood (27%) is enough to establish the diagnosis of CPI, because we ruled out any possibility of a different mechanism of transmission to the transplacentary route (e.g., vector transmission), as proposed by some authors [4]. Molecular diagnosis of infections offers distinct advantages over serological and microscopical diagnostic tests; however, these advantages are dependent on strict control in different steps such as collection and storage of samples, DNA isolation, DNA amplification, and analysis of results [37]. Samples analyzed generally had low levels of infection, which could affect the sensitivity and specificity of the qPCR used, and also could explain the inconsistencies between the results in maternal, placental, and cord blood.

The prevalence of CPI in this study, considering results of qPCR in cord blood and placenta simultaneously, was 19%, but with microscopy it was zero. Other studies carried out in the same region of Colombia (Northwestern Colombia) during 2005-2006 reported a prevalence of CPI by thick blood smear that ranged from 0 to 2.7% [11, 12]. In summary, using qPCR, the prevalence of plasmodial infection was 7 times higher than the prevalence detected by microscopy. Therefore, plasmodial infection during pregnancy is a serious public health problem in this region and the health authorities must give priority attention. A prospective multicenter study involving different ecoepidemiological areas with malaria transmission in Colombia is required to measure adequately the magnitude of the problem in each region.

Variation in the frequency of CPI is expected between studies, even within the same country or region, as observed in the literature. The prevalence of CPI data we found is higher than that obtained by averaging the three previous reports found, which is 1.8% for thick film [11–13]. This is likely related to maternal parasitemia and the transmission place. In previous studies, the presence of a higher level of transmission was clear, compared with that existent between 2010 and 2014, during which our work was executed. In this period, a control strategy in the region (Urabá-Altos Sinú/San Jorge-Bajo Cauca region) was developed, called “Malaria Project Colombia,” which led to a 75% reduction in malaria cases between 2011 and 2014.

The degree of parasitemia in the placenta is significantly associated with placental malaria infection [29, 38]; these data indicate the role of maternal immunity acquired during pregnancy specifically against* P. falciparum*.

In the placental histological findings, CPI was not associated with changes in any of the placental sectors assessed (decidua, villi, and intervillous space) except for edema in villi and the increase in the number of immune cells in decidua and villi. Perhaps the low frequency and the low number of placental damage instances during CPI were due to the low parasitemia in the mother and placenta.

The frequency of apoptotic cells in the placenta and the expression of FAS, COX1, COX2, HIF, VEGF, TNF, and TGF-b in placenta and mother were significantly higher when CPI was present. These results indicate that although CPI was asymptomatic and submicroscopic, apoptosis, inflammation, and hypoxia were observed. This suggests that the pathogenic role of these asymptomatic and submicroscopic infections seems to be beyond doubt and that such infections should not be considered unimportant. Similar alterations have been reported in other studies in the same area [13, 32, 33, 39].

We did not find changes or quantitative alterations related to the* Plasmodium* species, except for the expression levels of IFN-g (higher in* P. falciparum*) and TNF (higher in* P. vivax*) in the placenta. Although these are preliminary results, other studies reported similar changes or alterations in gestational malaria and placental malaria between these two species [11, 13, 32–34].

Okafor et al. studied the risk factors associated with congenital malaria in Enugu, Southeastern Nigeria. They said that, on univariate logistic regression (ULR) with the presence or absence of the congenital malaria as the dependent variable, six out of the 13 putative risk factors tested were statistically significant: low versus higher socioeconomic classes, low versus normal birth weight, positive malaria parasitemia in placental, mother, or umbilical cord, and parity of 0-1 compared with other parities [40]. The authors added that, on multivariate logistic regression, 3 out of the 6 factors that were significant on ULR remained significant: positive malaria parasitemia in placental or cord blood and parity [40].

According to Falade et al. (2007), spontaneous clearance of parasitemia occurred in 62% of neonates before day 2 and 34% were symptomatic within 3 days of birth. Neonates were monitored for clinical features attributable to malaria; all patients underwent follow-up with clinical and parasitological review on days 2, 3, 7, and 14 [3]. This clinical and parasitological surveillance for at least three days for infants is highly recommended [41].

There is a relationship between the histological changes in the placenta and birth weight, since, in acute and more chronic infections, there are a lower birth weight, a higher rate of anemia, and lower levels of hemoglobin, whereas acute infection produces increased risk of preterm delivery. In cases of submicroscopic infections, with no increased risk of low birth weight, there is no decrease in hemoglobin. In relation to the cytokines and their relationship with birth weight, it has been reported that, with higher levels of TNF, there are lower birth weight and premature births as a consequence of the restriction of intrauterine growth associated with inflammation [41].

## 5. Conclusions

Based on qPCR, the frequency of* Plasmodium* infection in maternal, placental, and umbilical cord blood was 39%, 51%, and 27%, respectively. The finding that* P. falciparum* causes 2 out of 3 cases of CPI is noteworthy, although in this area the overall prevalence is* P. vivax* (about 65%). All cases of CPI are submicroscopic and asymptomatic infection. These two characteristics are constant in plasmodiales infections during pregnancy in this Colombian region.

Perhaps this is the cause of the low lethality in the CPI study, as there was only one stillborn among 137 infants evaluated. The pathological effect of the CPI studied seems clear, since the CPI infants had body weight of less than 107 grams, compared with those who did not have CPI.

The histopathological alterations and the expression of hypoxia markers, inflammation markers, apoptosis markers, and cytokines were significantly higher when CPI was present and no significant difference was presented by species of* Plasmodium*.

The public health problem is evident and the problem of not having a routine diagnostic test with high sensitivity for use in endemic areas is also evident. These situations require that health authorities and health personnel be more strict and careful in implementing care protocols for pregnant women. It is essential to carry out the diagnostic test for malaria whenever a pregnant woman attends an antenatal clinic.

We consider it urgent to adopt the strategy of intermittent preventive treatment of pregnant women (IPT-g) with sulfadoxine-pyrimethamine (as they do in Africa), or better associated with amodiaquine to cover both* P. falciparum* and* P. vivax*.

## Figures and Tables

**Table 1:** Frequency of IMP-CM *P. falciparum* in some countries in Africa and in Colombia.

Site	CPI	Tested		Population	Ref.
Africa					

*Incidence*	(+)	*n*	*%*		
Ibadan, Nigeria	16	72	22,2	In deliveries screened	Sowunmi et al. (1996a)
Enugu, Ibadan, Kaduna, and Kwara, Nigeria	96	1875	5,1	Pairs mother-neonate	Falade et al. (2011)
*Mean*	*112*	*1947*	*5,8*		

*Prevalence*	(+)	*n*	*%*		
Lagos, Nigeria	16	104	5,3	Mothers attending the antenatal clinic	Mukhtar et al. (2006)
Lagos, Nigeria	14	100	14,0	Lagos University Teaching Hospital; following up: 28 days	Lesi et al. (2005)
Lagos, Nigeria	1	105	1,0	Parasitaemia (+); no disease	Lamikanra (1993)
Ibadan, Nigeria	11	77	14,0	11/77 = 14 among groups of low socioeconomic status	Olowu et al. (2000)
Ibadan, Nigeria	2	27	7,0	Among groups of middle to high socioeconomic status	Olowu et al. (2000)
Port Harcourt, Nigeria	19	26	73,1	University of Port Harcourt Teaching Hospital	Ununuma et al. (2014)
Port Harcourt, Nigeria	5	14	35,7	University of Port Harcourt Teaching Hospital	Nweneka et al. (2007)
Calabar, Nigeria	71	202	37,0	Among 202 neonates with suspected sepsis: 71/202 = 37%	Ekanem et al. (2008)
Enugu, Nigeria	203	625	32,5	University of Nigeria Teaching Hospital	Okafor et al. (2006)
Ile-Ife, Nigeria	65	120	54,2	2/65 had fever within 48 hours of life	Obiajunwa et al. (2005)
7 sub-Saharan sites^a^	7	100	7,0	Peripartum mothers and on cord blood; range: 0–23	Fischer (1997)
*Mean*	*414*	*1500*	*27,6*		
Southern Province of Zambia	19	65	29,0	Near-term newborns; Macha Hospital, in peak prevalence	Larkin and Thuma (1991)
Southwestern Cameroon	57	730	7,8	Mutengene Maternity Centre	Akum et al. (2005)
Malawi	8	131	6,0	Parasitaemia in cord blood by microscopy; 20% by PCR	Kamwendo et al. (2002)
Accra, Ghana, 2008	9	405	2,2	Untreated newborns <1 week of age; Korle-Bu Teaching Hospital	Enweronu-Laryea et al. (2013)
Accra, Ghana, 2010	0	467	0,0	Same as above; 12% by PCR	Enweronu-Laryea et al. (2013)
*Mean*	*93*	*1798*	*5,2*		

Colombia					

*Incidence*	(+)	*n*	*%*		
Turbo	3	351	0,85	Neonates in local hospitals and up to 30 days old	Franco et al. (1985)

*Prevalence*	(+)	*n*	*%*		
Arboletes, Necoclí, Turbo, and Carepa (Urabá zone)	5	183	2,7	Infants born at local hospitals	Carmona-Fonseca et al. (2009)
Arboletes, Necoclí, Turbo, and Carepa (Urabá zone)	2	79	2,5	Infants born at local hospitals; by nPCR: 13,0%	Campos et al. (2011)
Turbo and Puerto Libertador	0	121	0,0	Infants born at local hospitals; by nPCR: zero	Agudelo et al. (2013)
*Mean*	*7*	*383*	*1,8*		

^a^Numbers assigned by us based on percentage given by authors.

**Table 2:** Demographic and clinical characteristics of women and neonates studied, according to the presence or absence of plasmodial infection in cord blood.

Variable	Plasmodial infection in cord	*n*	Mean	SD^d^	SE^e^	95% CI mean Lower L; Upper L^f^		Min^g^	Max^g^	*p* (K-W)^h^
Age	No	93	22.78	6.29	0.65	21.49	24.08	14	42	0.535
	Yes	36	23.11	5.54	0.92	21.24	24.99	14	35	
Parity	No	91	1.75	2.06	0.22	1.32	2.18	0	9	0.239
	Yes	35	2.31	2.47	0.42	1.47	3.16	0	8	
Positive history of PM as medical history	No	27	28%							(*χ*^2^) 0.753
	Yes	9	25%							
Month of gestation when infection occurred^a^	No	24	5.75	2.55	0.52	4.68	6.82	2	9	1.000
	Yes	6	5.67	2.32	0.95	3.24	8.10	4	9	
Parasitemia at prenatal visit^b^	No	10	3053.30	3745.38	1184.39	374.01	5732.59	77	12000	1.000
	Yes	3	805.00	45.00	25.98	693.21	916.79	760	850	
Weeks of gestation at birth^c^	No	82	37.90	2.79	0.31	37.28	38.51	23	41	0.284
	Yes	35	37.47	2.72	0.46	36.53	38.40	27	40	
Maternal hemoglobin	No	71	11.114	1.669	0.198	10.719	11.509	7.2	18.3	0.139
	Yes	27	11.456	1.165	0.224	10.995	11.916	9.1	13.4	
Birth weight	No	100	3013.500	393.154	39.315	2935.490	3091.510	2230	4100	0.240
	Yes	36	2906.111	405.465	67.577	2768.922	3043.301	2150	3900	

^a^Number of months pregnant when gestational *Plasmodium* infection occurred.

^b^Parasitemia level found in prenatal consultation when the episode of gestational malaria happened.

^c^Number of weeks of gestation at birth.

^d^SD: standard deviation.

^e^SE: standard error of the mean.

^f^95% CI mean: confidence interval of 95% for the average; Lower L: lower limit; Upper L: upper limit.

^g^Min: minimum value; Max: maximum value.

^h^ *p*  (K-W) probability associated with the Kruskal-Wallis or chi-squared (*χ*^2^) test.

**Table:** (a) In cord-placental blood and cord-maternal blood

	Umbilical cord				Total
	None	*P. falciparum*	*P. vivax*	Both	
*Placenta*					
None	62	3	2	0	67
*P. falciparum*	18	18	0	0	36
*P. vivax*	18	1	7	0	26
Both	2	4	1	1	8
*p (X* ^2^ *) = 0.000*	*100*	*26*	*10*	*1*	*137* ^a^
*Mother*					
None	68	11	4	0	83
*P. falciparum*	12	14	0	0	26
*P. vivax*	20	1	6	0	27
Both	0	0	0	1	1
*p (X* ^2^ *) = 0.000*	*100*	*26*	*10*	*1*	*137* ^a^

^a^Positive cases: cord 27% (31/137), placenta 51% (70/137), and mother 39% (54/137).

**Table:** (b) In three compartments simultaneously

#	Umbilical cord blood	Placental blood	Maternal blood	*n* (%)
1	*P. falciparum*	*P. falciparum*	*P. falciparum*	13 (35)
2	*P. falciparum*	*P. falciparum*	None	5 (13)
3	*P. vivax*	*P. vivax*	*P. vivax*	4 (10)
4	*P. vivax*	*P. vivax*	None	3 (8)
5	*P. falciparum*	None	*P. falciparum*	1 (3)
6	*P. vivax*	None	*P. vivax*	1 (3)
7	Both	Both	Both	1 (3)
8	*P. falciparum*	Both	None	3 (8)
9	*P. vivax*	Both	*P. vivax*	1 (3)
10	*P. falciparum*	Both	*P. vivax*	1 (3)
11	*P. falciparum*	None	None	2 (5)
12	*P. vivax*	None	None	1 (3)
13	*P. falciparum*	*P. vivax*	None	1 (3)
Total				37 (100)

**Table:** (a) Prevalence of histologic event

Sector/event	*Plasmodium* in cord				*p* (*χ*^2^)
	No		Yes		
*Decidua*	*n = 100*	*%*	*n = 37*	*%*	
Immune cells	61	61	26	70	0.317
Atherosis	46	46	12	32	0.254
Necrosis	2	2	1	3	0.803
Abruptio	49	49	13	35	0.148

*Villi* ^a^					
Infarction	84	84	31	84	0.976
Edema	86	86	26	70	**0.034**

*IVS*					
Immune cells	100	100	100	100	1.000
Hemorrhage	95	95	36	97	0.560
Thrombosis	50	50	18	49	0.888
Calcifications	70	70	25	68	0.784

^a^In the villi, the following variables were also evaluated: (1) villi/field, (2) capillaries/villus, (3) immune cells, (4) fibrinoid deposits, and (5) syncytial knots. In all placentas, these variables were always present and therefore we do not apply the presentation in terms of present versus absent. The event is always present, either with or without the presence of *Plasmodium* infection, which varies in the magnitude of the event.

**Table:** (b) Quantitative comparison of the variables

	*Plasmodium*	Mean	SD	SE	*p* (M-W)
*Decidua*					
Immune cells	No	0.57	0.835	0.08	**0.001**
	Yes	1.21	1.116	0.183	
Atherosis	No	0.03	0.03	0.003	0.226
	Yes	0.02	0.03	0.005	
Necrosis	No	0.0005	0.003	0.0003	0.804
	Yes	0.0006	0.004	0.0007	
Abruptio	No	0.06	0.08	0.008	0.072
	Yes	0.03	0.07	0.01	

*Villi*					
Immune cells	No	0.70	0.515	0.05	***0.059***
	Yes	0.87	0.533	0.09	
Fibrinoid deposits	No	1.84	0.591	0.06	0.764
	Yes	1.86	0.671	0.110	
Syncytial knots	No	3.00	1.367	0.137	0.239
	Yes	2.72	1.181	0.194	
Villi/field	No	8.74	1.551	0.155	0.442
	Yes	8.50	1.872	0.308	
Capillaries/villus	No	3.91	1.168	0.117	0.589
	Yes	4.02	1.456	0.182	

*Intervillous space*					
Immune cells	No	2.29	1.458	0.146	0.100
	Yes	2.71	1.534	0.252	
Hemorrhage	No	0.36	0.206	0.02	0.639
	Yes	0.34	0.208	0.03	
Thrombi	No	0.03	0.06	0.006	0.838
	Yes	0.03	0.05	0.008	
Calcifications	No	0.21	0.374	0.04	0.761
	Yes	0.21	0.373	0.06	

**Table 5:** Expression of apoptosis, inflammation, and angiogenic markers, as well as pro- and anti-inflammatory cytokines in the placenta, according to the presence or absence of plasmodial infection in cord^a^.

Marker	Variable	Plasmodial infection in cord	Mean	SD	SE mean	95% CI mean Lower L; Upper L		Min	Max	*p* (M-W)
Apoptosis	Apoptosis (%)	No	56.4	13.8	1.86	52.7	60.2	39	81	**0.001**
		Yes	69.5	6.8	1.52	66.4	72.7	58	86	
	FAS^b^	No	4.3	1.2	0.17	4.0	4.6	2.0	6.0	**0.001**
		Yes	5.3	0.0	0.09	5.2	5.5	4.4	6.0	
	FASL	No	6.2	0.8	0.11	6.0	6.5	5.1	9.0	**0.009**
		Yes	5.8	0.4	0.08	5.6	5.9	5.1	6.6	

Inflammation	COX1	No	7.6	6.2	0.84	5.9	9.3	0.1	15.3	**0.000**
		Yes	13.7	1.8	0.41	12.8	14.5	10.5	16.5	
	COX2	No	3.8	3.0	0.41	3.0	4.7	0.1	9.0	**0.001**
		Yes	6.7	1.2	0.26	6.2	7.3	4.9	8.9	

Angiogenesis	HIF	No	0.9	0.5	0.06	0.8	1.0	0.0	2.2	**0.002**
		Yes	1.2	0.3	0.07	1.0	1.3	0.8	2.1	
	VEGF	No	0.9	0.3	0.04	0.9	1.0	0.1	1.4	**0.011**
		Yes	1.1	0.1	0.02	1.0	1.1	0.9	1.2	

Proinflammatory cytokines	IFN-g	No	5.9	4.2	0.57	4.7	7.0	1.1	13.1	**0.000**
		Yes	10.1	1.6	0.37	9.4	10.9	8.0	14.0	
	TNF	No	7.3	4.3	0.58	6.2	8.5	2.0	12.8	**0.047**
		Yes	10.7	0.8	0.18	10.4	11.1	9.0	11.8	

Anti-inflammatory cytokines	IL-2	No	1.2	0.4	0.05	1.1	1.3	0.4	2.3	0.075
		Yes	1.0	0.2	0.04	1.0	1.1	0.8	1.4	
	IL-4	No	1.4	0.3	0.05	1.3	1.5	0.0	2.4	0.181
		Yes	1.6	0.3	0.07	1.4	1.7	1.2	2.4	

Immunomodulatory cytokines	IL-10	No	2.7	1.5	0.20	2.3	3.1	0.1	5.0	0.064
		Yes	3.6	0.5	0.12	3.4	3.9	3.0	4.9	
	TGF-b	No	1.9	0.6	0.09	1.7	2.1	1.0	3.2	**0.002**
		Yes	2.4	0.4	0.08	2.2	2.6	1.6	3.0	

^a^Always *n* = 55 in IPM-MCN and *n* = 20 in IPC-MC (+).

^b^In all variables, measurement refers to relative quantitation in which the basal expression of a housekeeping gene was compared to the target gene according to the CT value of each gene. The graph reveals amplification (amplification plot). The value of CT has no units (expresses the ΔRn/cycle ratio).

**Table 6:** Expression of cytokines in maternal peripheral blood, according to the presence or absence of congenital infection^a^.

Marker	Cytokine	Plasmodial infection in cord	Mean	SD	SE mean	95% CI mean Lower L; Upper L		Min	Max	*p* (M-W)
Anti-inflammatory cytokines	IL-2^b^	No	2.3	0.5	0.07	2.1	2.4	1.1	2.9	**0.000**
		Yes	2.7	0.3	0.06	2.6	2.9	2.0	3.2	
	IL-4	No	1.4	0.2	0.03	1.3	1.4	1.0	1.9	0.557
		Yes	1.4	0.3	0.06	1.2	1.5	1.0	1.9	
Immunomodulatory cytokines	IL-10	No	2.2	1.3	0.18	1.9	2.6	0.1	4.0	**0.002**
		Yes	3.4	0.2	0.05	3.3	3.5	3.0	3.9	
	TGF-b	No	1.0	0.5	0.06	0.9	1.1	0.1	1.9	**0.027**
		Yes	1.3	0.2	0.05	1.2	1.4	0.9	1.8	
Proinflammatory cytokines	IFN-g	No	5.5	2.6	0.35	4.8	6.2	2.1	9.4	**0.000**
		Yes	8.2	1.1	0.24	7.7	8.7	6.7	10.7	
	TNF	No	6.0	2.9	0.39	5.2	6.7	2.1	9.7	**0.010**
		Yes	8.4	0.4	0.10	8.2	8.6	7.4	9.6	

^a^Always *n* = 55 in IPM-MCN and *n* = 20 in IPC-MC (+).

^b^In all variables, measurement refers to relative quantitation in which the basal expression of a housekeeping gene is compared and evaluated for the problem gene, according to the CT value of each gene. The graph reveals amplification (amplification plot). The value of CT has no units (expresses the ΔRn/cycle ratio).
